# Human Improvability

**Published:** 1932

**Authors:** Charles S. Myers

**Affiliations:** Principal of the National Institute of Industrial Psychology, etc.


### HUMAN IMPROVABILITY.

BY

Charles S. Myers, C.B.E., F.R.S., M.A., M.D., D.Sc.,

*Principal of the National Institute of Industrial Psychology, etc.*

---

The problem of human improvability is as interesting for discussion as it is difficult of solution. It is essentially a problem of relatively recent origin. For the notion of progress was only generally entertained when that of evolution came to be accepted. In ancient times mankind was conceived as tossed hither and thither in the grip of fate or fortune ; while in medieval times mankind was regarded as being perpetually steeped in sin until the dawn of a Messianic millennium.

Perhaps the greatest difficulty which confronts us in approaching this problem is that of defining improvement. Unless we can clearly determine whether any change makes for improvement, how can we hope to ascertain whether or not human improvability is possible ?

I think that at the outset we may advantageously distinguish "progress" from "improvement." Every step in evolution, *i.e.* every change making for increased differentiation of function, increased co-ordination of parts and increased integration of previously independent units, may be accepted as progress.

But improvement implies something more—namely, "betterment." Unlike progress, improvement is bereft of any scientific, objective criterion. We have to judge of improvement by subjective, ethical criteria. We have to ask ourselves whether the change leads not merely from a lower to a higher plane of organization, but also from a lower towards a higher ideal. Improvement has thus a significance relative to the user of the term. What one person will regard as an improvement will not necessarily be so regarded by another. We have also to consider each change, not in isolation, but in relation to all other relevant changes, and in relation to the total environment, if we are to regard it as an improvement.

The prevailing current biological view is that all changes in living form and function are evoked by accident and are perpetuated by heredity and by their suitability to the environment. On this view we might regard improvement as consisting in a more perfect adaptation or adjustment to our environment, physical and social. Certainly in the conquest of disease, in the discovery and introduction of more highly sanitary conditions, we of the modern Western civilization may, in this sense of the word, be said to be "improving." But improvement from this aspect may be accompanied, as we are all aware, by deterioration from another, *e.g.* by the increased survival of the less fit and the unfit. Yet here once again we have to turn to the reverse aspect: the greater care of the infirm may imply a social, moral improvement, in developing the "instinct"—shall we call it ?—of protecting the weak and in reducing the sway of ruthless selfishness and of brute force.

This notion of social improvement leads us to yet another conception of improvement—as measured by the extent to which we carry out in practice our moral rules and ideals. Codes of morality may vary from age to age, according to social environment and other factors. May not the true criterion of social improvement be not merely the nobility of our moral ideals, but also, and rather, the extent to which we succeed in putting them into actual practice ?

If, finally, we bring together two of these different meanings of improvement which we have been considering—the idea of improved social evolution on the one hand and that of improved adaptation to environment on the other—we are confronted with the following conceivable situation. There may gradually be evolving a state of society so complex, so exacting, so strenuous and so full of conflicts that man may be unable to adapt himself to it as he did in the easier, slower life of centuries ago. He may ultimately be destroyed by the weight of his social environment, just as perhaps the huge dinosaurus perished through its own unwieldiness. So far from progressing and improving, man may be forced to revert to simpler conditions for his own salvation.

It is possible, however, to hold quite another view of the determining conditions of progress and improvement. Many find it difficult to believe that the evolution of species has occurred through purely accidental variations in the germ plasm and in consequent structural and functional progress, better fitted to life's environment. To them it seems as necessary to invoke the presence of some unknown direction and purpose or end throughout evolutionary progress as it is to invoke direction and purpose or end in accounting for the composition of one of Shakespeare's plays or of Titian's pictures. The creation of new forms of organisms seems to them to be as impossibly accidental as the creation of masterpieces of art or as the discoveries in physical or biological science. On this view some unknown direction, purpose, or end is inherent in the history of the universe. It is a view which almost inevitably leads to a belief in general, and particularly in human, improvability.

But such a faith, however robustly held, is hardly adequate for a satisfactory solution of this problem, especially in a scientific gathering like that which I am now addressing. I think that you may be interested in hearing an account of my own experiences among primitive peoples in relation to the problem of human improvability. If we consider the most primitive peoples alive to-day, we cannot fail to be struck with the elaborate social institutions and regulations governing and restricting their life and conduct. The old idea we used to entertain of the greater freedom of the savage compared with civilized man turns out to be false. No one among ourselves is so fettered in his actions as primitive man by the dictates and authority of social and religious tradition and custom. Our liberation from such of them as are now judged useless or harmful, and have vanished through disuse, is surely one of the improvements effected by modern civilization.

Over thirty years ago I took part in an anthropological expedition to the islands of the Torres Straits, lying north of Australia between it and New

Guinea, and to Sarawak in Borneo, where we made a study *inter alia* of the mental powers of these peoples. The differences we found between them and civilized peoples as regards intelligence did not impress us as being appreciable. In those days intelligence tests were not available. But even if they had been in vogue they would have proved useless, for the results of intelligence tests can only be compared when they have been applied to individuals who have been born into and have grown up in the same social environment.

I have been told that some years ago the Americans attempted to prove the inferior intelligence of the Japanese by applying American tests of intelligence to Japanese children in Hawaii. The Hawaiian Japanese retorted by framing and standardizing tests of intelligence suited to Japanese, instead of to American culture and civilization. By applying these tests to American and Japanese children, they had no difficulty in proving that the American intelligence was inferior to the Japanese. Even within the United Kingdom the same intelligence tests are not comparable, say, between England and Scotland. So much depends on conditions of environment, education, etc.

Nor would the results of any tests we may devise to-day for intelligence be comparable with the results of the same tests applied to the same community one hundred years ago or one hundred years hence. Each generation has a language of its own, an education of its own, an environment of its own. And as no tests can be devised which are independent at once of language, of knowledge and of manual actions indicative of the exercise of intelligence, we are powerless to determine by scientific methods whether intelligence is improving within the same community from one century to another, or whether it is at the present day higher in one community, nation, or race than in another.

Let us return now to our Torres Straits islanders. This expedition of which I was a member examined *inter alia* the differences between the natives of the Torres Straits and Sarawak and peoples of our own civilization as regards sensory acuity—keenness of sight, hearing and smell, for instance, and sensitivity to pain. These (with the exception of the last) turned out to be relatively slight. The wonderful stories brought back by travellers as to the extraordinary visual powers of the savage seem attributable merely to his far more intimate previous knowledge, which enables him to interpret dimly-seen objects with a correctness which seems marvellous to the less expert European stranger. On Murray Island in the Torres Straits lived one white man, a Scottish school teacher, who gave instruction to the native children. In arithmetic I found him teaching them such now antiquated subjects as "practice" and "parcels." He told me that his pupils did rather better in arithmetic than corresponding children in our own country, and this despite the fact that in their own language the Murray islanders possessed only two words for number—"netat" for "one," and "naes" for "two." "Three" was expressed by "netat-naes" (one and two), four by "naes-naes" (two and two).

Such results may make us at first sight, at least, wonder whether savage peoples would not reach the mental levels of more civilized people if only they were born in the latter's civilization. For there can be no doubt that both animals and plants may quickly alter when removed to a new environment, and that there is a wider range of possibilities than has been hitherto generally supposed in the development of what is inherited by the organism, the precise nature of that development being determined by the nature of the environment to which it is subjected. Changes in food, soil, temperature, humidity and the like affect the final result of an organism's physical development. May we not suppose also that man's mental and moral development depends not so much, or at all events not merely, upon what is inherited, as upon the relation between that inheritance and the physical and social environment in which the individual grows up? How often do we ascribe the moral defects and delinquency of a young person to the fact that he never had a fair chance of living decently! May we not entertain the view that man's improvement is largely due merely to a certain innate improvability, the precise expression of which in turn depends on the environment in which he grows up?

During a subsequent visit to South and East Africa I had the opportunity of several talks with Europeans whose lives had been largely spent in teaching the coloured races there. With surprising unanimity their opinion was that, given equal opportunities, their coloured pupils promised to equal the achievements of any white population. They were vigorous in their denial that there was one education needed for the negro, another for the European. For them negro and European possessed equal mental capacity and equal powers of achievement, given equal opportunities of development and environment.

Against this view must be opposed the oft-quoted experience as regards the negroes in the United States. Despite several generations of education, not a single pure-blooded negro has achieved anything approaching first-class greatness, so it has been stated, judged from the white man's standards. Booker Washington and other similarly eminent coloured individuals have had white blood in their veins. To this, however, it may be objected, first, that there are so many negroes in the United States who are not pure, and consequently that we should not be surprised that those who succeed are not pure; and in the second place, that the environment in which they grow up and are educated fails to give them an opportunity equal to that enjoyed by white persons. They are taught by less efficient, often coloured teachers, grow up amid negro traditions and in negro society, and are regarded with contempt, aversion and ostracism by those whose civilization and culture they are expected to adopt.

On the other hand, the force of heredity and the impossibility of radical change in what is inherited must not be neglected. Nothing will stop true genius or the really criminal mind from asserting itself, whatever be the environment against which it may have to struggle. This is as certain as the fact that the very best education is powerless to change the innately mental defective into a person of even moderate intelligence. Why, then—we may well ask—is it that despite all the disadvantages under which he labours, a pure-blooded negro has not occasionally arisen who has equalled the highest genius of the white race in scientific discovery or in artistic creation ? Are we not bound to conclude that, however nearly alike may be the average abilities of two such different races, they differ, at all events in the extent of exceptional ability or in the temperament and character which permits of the best use of their ability ?

Long-standing differences of climate are doubtless largely responsible for the differences in colour between the black and white man and for the other physical and the temperamental differences between them, and between one European nation and another, and for the differences between their social institutions and culture. Can we, therefore, expect a relatively quick adaptation of a member of the lower races or of any other civilization to our own civilization, any more than we can expect a rapid change of his skin colour from black or brown to white ?

On the other hand, why should one primitive race or people remain apparently stagnant or show decadence in social conditions, whilst another steadily develops a civilization rich in scientific discovery and in general cultural progress ? Must this not be largely due to the influence of changes of climate and other features of environment in producing, selecting and stimulating those most fitted to contribute to social progress, especially by developing such internal secretions and consequently such temperamental, intellectual and moral characters as are best suited for initiating and accepting such advance ?

For my part, I cannot but believe that there are profound racial differences which have thus produced, and been produced by, cultural differences, and that these racial differences will persist long after attempts have been made, as modern civilization is now attempting, to bring all races under the same social environment. And I believe that these differences between white and coloured peoples constitute generally an improvement in the former, in so far (according to our original definition) as they relate to a higher culture and to a higher moral.

Let us now turn to another aspect of the problem, the changes occurring in our own race and civilization. We are often told that the present age, so far from being one of improvement, is an age of degeneracy, or that it is at all events an age which should be replaced —and replaced profitably—by some previous one. For my part I see much confusion of thought and personal prejudice in such a view. Transitory reverses in moral conduct are here regarded as evidence of permanent deterioration. The present lessened prospects, comfort and happiness of those who previously enjoyed light taxation, loyal and contented servants, large houses and estates, naturally blind them to improvements occurring among the large masses of our modern people. They think only of the vulgar manners and of the refusal to shoulder their own old responsibilities which they see (temporarily, as I maintain) among the *nouveaux riches* who have supplanted them.

The good old days, like the good old Christmas weather, must be regarded largely as a delusion. Neither will withstand any approach to exact inquiry. The monotony involved in modern industrial conditions and the evils of mass production are largely exaggerated, and, indeed, are relatively negligible in comparison with their compensatory advantages. Compare the conditions under which manufacture was once conducted in insanitary cottage homes with the present conditions of the most progressive, well-lighted factories, where welfare work and the investigations of the industrial psychologist and physiologist are in full swing—all directed to ensuring the greater physical and mental comfort of the worker, to reducing needless worry, irritation, boredom and strain, and to placing the worker in that kind of occupation for which he is mentally and physically best suited. Consider what mass production has achieved in enabling the standard of living of the masses to be raised, in providing them with shorter hours of work and with recreations (*e.g.* the wireless, the gramophone, the film, the charabanc, the motor cycle)—recreations far in advance of those which the so-called working classes of bygone generations could afford, desire, or even conceive for themselves.

True, it is said, and perhaps some of you will insist, that mass production and mass education destroy individuality and reduce us all to a common standard of uniform inferiority. But I maintain that this view is—in the long run—a mistaken one. Not long ago the maker of some of the finest modern tapestries now produced in this country told me that his designs were frequently being imitated in Germany and sold there cheaply as copies of his own designs ; but to my surprise he added that these advertised imitations really helped the sale of his own choice products, inasmuch as whenever a German had acquired enough wealth he preferred to buy the genuine British article in place of the cheaper, inferior mass-production copy of it already in his possession.

So it is, I hold, with the mass production of music by gramophone records or by wireless. The concert halls have not thereby suffered: that is generally admitted. You may not infrequently hear the street-boys of to-day whistling high-class music. Not long ago I heard a young barman at a small remote country inn singing an air from one of Saint-Saëns's operas. And when I asked him how he had come to learn it, he told me that he possessed a gramophone record of it. So it is too, I believe, with the cinema films; they offer an inducement to those of the working classes who are endowed with the best taste to exchange mechanized for living artistic productions and to visit good plays at the theatre.

Compare these mechanized products, however deplorable they often are, with the recreations available for the bulk of our people two or three generations ago. Remember, too, that good taste abounds in every social stratum, as in every level of civilization, awaiting only encouragement for its exercise. Many years ago I used occasionally to help the late Canon Barnett by attendance at his annual exhibition of modern paintings in Whitechapel; and almost invariably I observed the largest crowds of these East-End workers congregating around the best pictures, *e.g.* those of Watts, Millais and other first-rate artists of the day. Only a short while ago I heard of a poor working lad who was so impressed with some Bach music which had been played to him and his friends that he asked whether they could not themselves form an orchestra in order to play Bach music.

Mass production may produce temporary ill-effects ; it calls, for instance, temporarily in factories for armies of machine-feeders engaged on the most monotonous tasks ; and by replacing much human labour by machinery it increases unemployment temporarily. But presently production is cheapened ; the demand for the product is increased ; and the number of workers needed may rise by leaps and bounds. And presently the dull work of machine-feeding is itself performed mechanically, and the demand increases for a larger supply of machine-minders—*skilled* workers to look after the increasing number of machines. Ultimately more machinery means a greater demand for skilled workers and the abolition of severe physical or tedious labour of a type that is fast becoming regarded as unfit for civilized humanity. So it is, too, with the mass production of clothes. First it raises the standard of living, enabling those to buy articles who could never before have afforded them. Next it enables those possessing taste and an adequate income to demand articles of better manufacture, greater individuality and artistic merit, who in former days would never have had the opportunity of doing so.

The same holds for the mass provision of educational facilities. These are at present far from perfect, for the existence and the demands of individual mental differences in the general community have not hitherto been adequately recognized in our schemes of education. Yet who but the incompetent, who realizes that he has no right to his own social position, feels insecure in it, and wishes, therefore, to keep those born within a lower social stratum " in their right place"—who else can doubt the ultimate value of systems of more widely-spread, better education, if only it be properly and variously adapted to the mental abilities of the recipients? Wild, ill-considered, revolutionary ideas must arise in the early phase of the education of any mass community—just as they arise during the youth of any individual of ability receiving the very best conceivable education. They are indicative merely of commencing thought and liberty of thought.

The present age is often designated as irreligious, but in reality there was never so much thought so widely given to religious problems as at the present day. What has diminished is only the servile, thoughtless adherence to long-accepted religious formulæ, dogmas and practices.

No, I firmly believe that in conduct, in sympathy for our fellows, in refinement, in self-control, in grade and independence of thought, and in ideas of social service we are as a community, as a civilization, definitely improving, despite certain set-backs which are, to my mind, merely of a temporary nature, comparable to the transitory, depressing effects of a medicinal remedy which ultimately leads to a vast improvement in physical health. One has also to take into account certain changes in fashion which, whether for the better or for the worse, are not essential or permanent features of our mental and moral progress. Further, we have to recognize that changes for the better can never be wholly free from accompanying changes in certain other directions for the worse. We have also to recognize that, deeply as we may regret the passing of certain attractive features of by-gone days, we cannot reverse the march of events, any more than we can hope to give up machinery and revert to the hand-manufacture of articles which was only possible when our population was smaller and the masses of them had to be content with far fewer demands and with a far lower standard of living.

Never, as to-day, has each of us approached to so full a knowledge of himself and to so far better an appreciation of the standpoint, the ideas and the feelings of his fellows. The desire and the readiness for fighting between classes, between capital and labour, are perceptibly on the decrease, just as they are between at least the older European nations. Fighting is but the crudest, most primitive form of competition. The total abolition of competition, in its more advanced form at least, is at present unthinkable and undesirable. It is only wasteful, extravagant competition that needs to be suppressed and tends already to be disappearing, alike nationally and internationally by what is called industrial rationalization.

Must we infer physical and mental deterioration if, as individuals and as a nation, we abandon the more primitive forms of struggle for existence ? If deterioration occurs in certain directions, are not improvements equally inevitable in others ? And if, returning to our opening considerations, we ask how far these are real improvements in the nature of man, and how far they are due to the response of man to improvements in his environment, and would disappear if man were transplanted to a lower civilization—if, that is to say, we ask whether man has undergone any intrinsic improvement, and if so in what directions and how far—we may, I think, indicate our general position in the following conclusions :—

(*a*) That in many respects we are vastly improving.

(*b*) That in many respects, perhaps in all respects within a particular race or people, these improvements do not arise broadly and directly from the fact that most members of that race or people are innately improving, but rather from the fact that the social heritage, the civilization into which they are born is improving.

(*c*) That the causes of improvement in the social heritage are ultimately due to improvements in a few "leading" individuals themselves for which their physical environment is, in part, responsible.

